# Individual differences in EEG correlates of recognition memory due to DAT polymorphisms

**DOI:** 10.1002/brb3.870

**Published:** 2017-11-10

**Authors:** Paolo Medrano, Erika Nyhus, Andrew Smolen, Tim Curran, Robert S. Ross

**Affiliations:** ^1^ Psychology Department University of New Hampshire Durham NH USA; ^2^ Department of Psychology and Program in Neuroscience Bowdoin College Brunswick ME USA; ^3^ Institute for Behavioral Genetics University of Colorado Boulder Boulder CO USA; ^4^ Department of Psychology and Neuroscience University of Colorado Boulder Boulder CO USA; ^5^ Neuroscience and Behavior Program University of New Hampshire Durham NH USA

**Keywords:** DAT, item memory, old/new effect, oscillations

## Abstract

**Introduction:**

Although previous research suggests that genetic variation in dopaminergic genes may affect recognition memory, the role dopamine transporter expression may have on the behavioral and EEG correlates of recognition memory has not been well established.

**Objectives:**

The study aims to reveal how individual differences in dopaminergic functioning due to genetic variations in the dopamine transporter gene influences behavioral and EEG correlates of recognition memory.

**Methods:**

Fifty‐eight participants performed an item recognition task. Participants were asked to retrieve 200 previously presented words while brain activity was recorded with EEG. Regions of interest were established in scalp locations associated with recognition memory. Mean ERP amplitudes and event‐related spectral perturbations when correctly remembering old items (hits) and recognizing new items (correct rejections) were compared as a function of dopamine transporter group.

**Results:**

Participants in the dopamine transporter group that codes for increased dopamine transporter expression (10/10 homozygotes) display slower reaction times compared to participants in the dopamine transporter group associated with the expression of fewer dopamine transporters (9R‐carriers). 10/10 homozygotes further displayed differences in ERP and oscillatory activity compared to 9R‐carriers. 10/10 homozygotes fail to display the left parietal old/new effect, an ERP signature of recognition memory associated with the amount of information retrieved. 10/10 homozygotes also displayed greater decreases of alpha and beta oscillatory activity during item memory retrieval compared to 9R‐carriers.

**Conclusion:**

Compared to 9R‐carriers, 10/10 homozygotes display slower hit and correct rejection reaction times, an absence of the left parietal old/new effect, and greater decreases in alpha and beta oscillatory activity during recognition memory. These results suggest that dopamine transporter polymorphisms influence recognition memory.

## 1 INTRODUCTION

Recognition memory refers to an individual's ability to correctly identify previously encountered stimuli and is influenced by genetic variation in dopaminergic genes (Jocham et al., 2009; Li et al., 2013; Papassotiropoulos & Quervain, 2011; Papenberg et al., 2014; Schott et al., 2006; Takahashi et al., 2007). Specifically, altered dopamine transporter expression resulting from the dopamine transporter gene (DAT) affects behavioral and neuroimaging correlates of long‐term memory processes (Li et al., 2013; Schott et al., 2006). The differential dopaminergic neurotransmission that results from the varied expression of DAT may alter the course of recognition memory retrieval processes, resulting in differences between individuals’ ability in identifying previously encountered stimuli. However, it is currently unclear whether DAT genetic variation affects an individuals’ recognition memory through processes associated with the retrieval of information itself or through cognitive control processes that serve to monitor and evaluate retrieved information. Therefore, this study uses electroencephalography (EEG) in combination with genetic data collection to show how dopaminergic transporter polymorphisms may alter the processes underlying memory retrieval during recognition memory.

EEG studies of memory have identified four distinct event‐related potential (ERP) signatures associated with recognition: the early old/new effect (FN400), the parietal old/new effect, the late frontal old/new effect, and the late posterior negativity. The FN400 and left parietal old/new effects are ERP correlates that index memory processing (Curran, 2000; Curran & Hancock, 2007; Donaldson & Rugg, 1998; Friedman & Johnson, 2000; Rugg & Curran, 2007; Rugg et al., 1998; Vilberg, Moosavi, & Rugg, 2006; Vilberg & Rugg, 2007; Wilding, 2000), whereas the late frontal old/new effect (1,000–1,500 ms) and late posterior negativity (LPN) are EEG correlates associated with cognitive control. Cognitive control may aid memory retrieval through the activation of processes that retrieve associated contextual details for further evaluation or monitor and evaluate the retrieved information (Friedman & Johnson, 2000; Hayama, Johnson, & Rugg, 2008; Hayama & Rugg, 2009; Johansson & Mecklinger, 2003; Mecklinger, Rosburg, & Johansson, 2016; Rugg & Wilding, 2000). Dopamine has been shown to affect processes of both memory retrieval (Apitz & Bunzeck, 2013; Bunzeck, Doeller, Fuentemilla, Dolan, & Duzel, 2009; Eckart & Bunzeck, 2013) and cognitive control (Cools, 2008; van Schouwenburg, Aarts, & Cools, 2010), and previous research has linked DAT polymorphisms with variations in memory performance (Li et al., 2013; Schott et al., 2006). This study utilizes these four well‐known ERP signatures of recognition in order to discern the effects that DAT expression has on recognition memory.

Alongside ERP correlates of recognition memory, brain oscillatory activity has been associated with recognition memory. Brain oscillations are rhythmic fluctuations in electrical charge, which are related to local and network neural communication and integration (Buzsáki, 2006; Fries, 2005). Previous research has associated activity in the theta (4–8 Hz), alpha (8–13 Hz), beta (13–30 Hz), and gamma (30–100 Hz) frequency bands to memory processes (Addante, Watrous, Yonelinas, Ekstrom, & Ranganath, 2011; Axmacher, Mormann, Fernandez, Elger, & Fell, 2006; Burke et al., 2014; Fell, Ludowig, Rosburg, Axmacher, & Elger, 2008; Fellner, Bäuml, & Hanslmayr, 2013; Hanslmayr, Spitzer, & Bauml, 2009; Hanslmayr et al., 2011; Hasselmo & Stern, 2014; Heusser, Poeppel, Ezzyat, & Davachi, 2016; Jacobs, Hwang, Curran, & Kahana, 2006; Klimesch, Doppelmayr, Russegger, & Pachinger, 1996; Lega, Jacobs, & Kahana, 2012; Nyhus & Curran, 2010; Osipova et al., 2006; Sederberg et al., 2007; Staudigl & Hanslmayr, 2013; Summerfield & Mangels, 2005; Waldhauser, Johansson, & Hanslmayr, 2012; Watrous, Tandon, Conner, Pieters, & Ekstrom, 2013; Weiss & Rappelsberger, 2000). Specifically, increases in theta and gamma synchrony may serve to coordinate processes of synaptic plasticity and memory reactivation (Nyhus & Curran, 2010), whereas desynchronization in the alpha and beta frequency ranges may play a role in memory by desynchronizing local neural assemblies, allowing for the transmission of more information during both encoding and retrieval processes (Hanslmayr, Staudigl, & Fellner, 2012). Dopamine affects memory and executive functioning related oscillatory activity (Benchenane, Tiesinga, & Battaglia, 2011; Benchenane et al., 2010; Eckart, Fuentemilla, Bauch, & Bunzeck, 2014), and by determining how *DAT* influences both ERP and oscillatory correlates of recognition memory, our study may reveal how individual differences in dopaminergic functioning changes recognition memory.

Differences in recognition memory, ERP old/new effects, and oscillatory activity associated with recognition memory were examined between participants homozygous for the 10‐repeat (10R) VNTR of the dopamine transporter gene and participants possessing a copy of the 9 (9R) repeat VNTR during an item memory task. Previous research suggests that decreased synaptic dopamine clearance is beneficial for memory (Li et al., 2013; Schott et al., 2006). Therefore, we hypothesize that participants homozygous for the 10R‐allele, which results in increased DAT expression (Fuke et al., 2001) and increased dopaminergic clearance (Heinz et al., 2000) will display impaired item memory performance, alongside diminished ERP and oscillatory correlates of memory compared to participants that possess a 9R‐allele.

## 2 MATERIALS AND METHODS

### 2.1 Participants

Seventy‐six right handed participants from the University of Colorado Boulder community volunteered to participate in this study. All participants gave informed consent in accordance with the Institutional Review Board of the University of Colorado Boulder. Sixteen participants were removed from the study for various reasons. Four participants failed to complete the entirety of the study, and three were removed for technical reasons. Nine participants were removed due to excessive noise in the EEG recordings, including excessive blinking (*n* = 3), the required use of excessive channel interpolation (*n* = 2), the lack of 20 good hit and correct rejection epochs for comparison postartifact detection (*n* = 3), and the lack of adequate behavioral performance (*n* = 1). The removal of these participants resulted in a total of 60 participants aged 18–29 (mean ± standard deviation, 20.7 ± 2.59 years old; 27 females, 33 males) for analysis. *DAT* groups were split according to whether the variable nucleotide tandem repeat (VNTR) sequence that influences DAT expression repeated 9 or 10 times (Fuke et al., 2001). Of the 60 participants participating in the study, two participants (one male, one female) possessed a DAT genotype that failed to fit in either the established 9R‐carrier or 10/10 homozygous group and were not included for *DAT* group analysis. The 31 participants that were heterozygous or homozygous for the 9R‐allele (i.e., 9/9 or 9/10) were placed in one group (14 female, 17 male), whereas 27 participants (12 female, 15 male) homozygous for the 10R VNTR were placed in the other.

### 2.2 Stimuli

**Figure 1:**
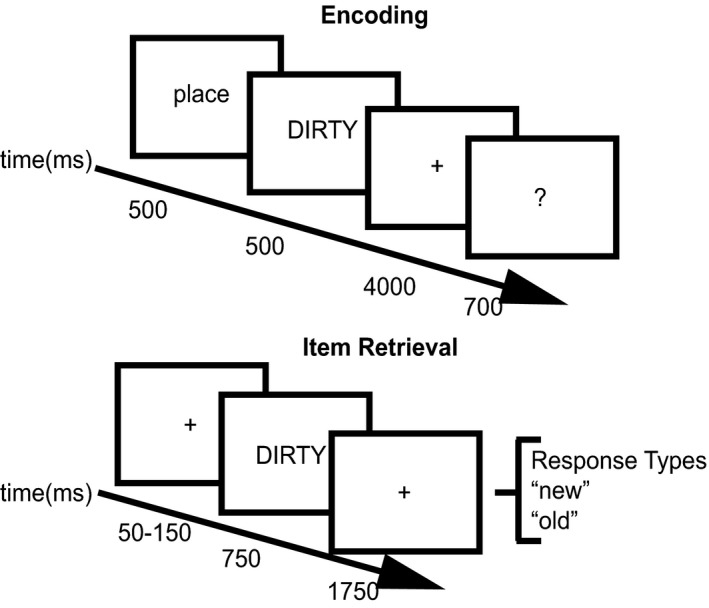
Behavioral paradigm used during recognition memory task. During the encoding phase, participants were given a place or pleasantness cue for 500 ms indicating the task to use during encoding. Following this cue, an adjective was presented for 500 ms. Participants were given 4,000 ms to perform the encoding task and then were asked to rate how successfully they were performing the task. The bottom panel represents the retrieval phase where EEG recordings took place. A variable duration fixation cue was presented for 50–150 ms followed by an adjective for 750 ms and a fixation cross for 1,750 ms. Participants could respond at any time after presentation of the adjective with one of two choices, “new” or “old”

Eight hundred and fifteen adjectives were used as stimuli. The Kucera and Francis (1967) word norms were used for the selection of adjectives in the study. The words were presented to the participants in white uppercase letters in the center of the screen on a 26 in LCD computer screen with a black background at a visual angle of 2.3° (Figure 1). The average written frequency (kfreq) of all the adjectives used in the study was 34.86 and the average number of letters per word was 6.93. The average kfreq across the counterbalanced lists ranged from 34.19 to 35.93 and the average number of letters across counterbalanced lists ranged from 6.87 to 7.00 and the kfreq and number of letters did not differ between lists.

### 2.3 Task

Participants performed an item memory task during one study session, and a separate, source memory task was performed during a separate study session on a different day. The source memory data will be presented elsewhere. For the item memory task, participants were presented a list of words and asked to encode them during the study phase. In order to familiarize participants with the task, participants first underwent a short practice block before being asked to encode words in the study block. During this practice block, participants were given instructions and studied 10 words in order to familiarize them with the task. Upon completion of the practice block, the study block began. The study block consisted of 204 words, with two words at the beginning and two words at the end of the study block acting as primacy and recency buffers. During the study block, participants were instructed to associate half of the words with the mental image of a place and the other half were asked to make a pleasantness rating (Davachi, Mitchell, & Wagner, 2003; Kahn, Davachi, & Wagner, 2004). A place or pleasantness cue was presented for 500 ms prior to adjective presentation, which lasted for 500 ms. A fixation cross was presented for 4,000 ms after adjective presentation to allow participants to perform the encoding task. Upon completion of the encoding period, a question mark popped up on the screen for 700 ms, a period in which participants were instructed to rate the degree to which they successfully encoded the adjective (Figure 1). Participants rated their performance by pressing one of four buttons: (1) unsuccessful; (2) partially successful; (3) successful with effort; (4) successful with ease.

Following the study block, item memory retrieval was tested while participants underwent EEG recording. Participants were fitted with a 128 channel Hydrocel Geodesic Sensor Net connected to an AC‐coupled high input impedance amplifier (200 MΩ, Net Amps TM, Electrical Geodesics Inc., Eugene, OR). Amplified analog voltages (0.1–100 Hz bandpass) were digitized at 250 Hz. Individual sensors were adjusted until impedances were less than 50 kΩ. Participants were given a 15‐word practice test block prior to beginning the retrieval task. Approximately 30 min passed between the conclusion of the encoding phase and the beginning of the retrieval phase of the study. Participants viewed 480 words during the item retrieval test: 200 previously studied words, 200 new words, and 80 words serving as buffers. The adjectives were presented in blocks of 24, with two words at the beginning and end of each block serving as primacy and recency buffers. Twenty test blocks were used to test item memory retrieval. For each presented adjective, there was an initial variable fixation period of 50–150 ms, followed by the test word for 750 ms and an additional fixation period of 1,750 ms. Participants were permitted to respond upon word presentation. To respond, participants used the index fingers of both hands and pressed one key for an old (previously studied word) and another key for a new word. Following their response, participants used the index and middle finger of one hand and the index finger of their other hand to provide information regarding the degree of confidence of their answer. One key was pressed for “surely,” one key was pressed for “likely,” and another key was pressed for “maybe.” EEG data, accuracy data, and reaction time (RT) data were collected as participants completed the task.

### 2.4 ERP preprocessing

For ERP preprocessing, EEGLAB (Delorme & Makeig, 2004; RRID: SCR_007292) and ERPLAB (Lopez‐Calderon & Luck, 2014; RRID: SCR_009574) were used. Before data preprocessing, channels with excessive noise were identified via visual inspection and interpolated using spherical spline interpolation. Two participants that required the interpolation of more than five channels (4%) were not included in the final data analysis. Data processing included filtering the data from 0.1 to 40 Hz, rereferencing to the average signal, separating the data into epochs, and artifact rejection. The data were epoched into periods 800 ms prestimulus presentation to 1,500 ms poststimulus presentation (−800 to 1,500 ms). Epochs were sorted into bins according to their response type (hits and correct rejections). Correctly remembering an item as one previously encountered constituted a hit, whereas correctly indicating that a word had never been seen before constituted a correct rejection (CR). Artifact rejection was accomplished with an automated moving window search procedure where changes of 100 μV were marked for rejection in 50 ms bins of 100 ms length. A threshold of 20 clean, artifact free epochs for each type of response (hit and correct rejection) postartifact rejection was established for participant inclusion in data analysis. Analysis of the ERP datasets that met this threshold revealed an average of 111.84 ± 39.02 hits epochs (10/10 homozygotes: 117.96 ± 39.16; 9R‐carriers: 105.42 ± 37.59) and 104.67 ± 35.69 correct rejection epochs (10/10 homozygotes: 103.26 ± 35.51; 9R‐carriers: 105.45 ± 36.10). To ensure no significant differences between the number of hits and correct rejection trials within DAT groups were present, a pair of independent samples *t* tests were conducted. Results indicated that there were no significant differences between the number of hits and correct rejection trials in 10/10 homozygotes (*t*_56_ = 1.20, *p* = .22) or 9R‐carriers (*t*_56_ = 0.23, *p* = .52).

### 2.5 ERP regions of interest

**Figure 2:**
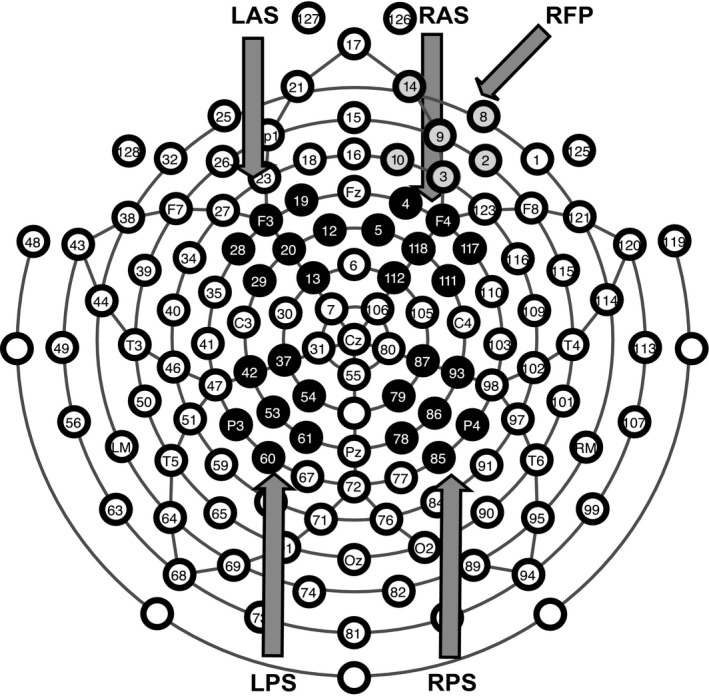
Regions of interest for ERP analyses. Electrode montage representing the location of all 128 electrodes. Black and gray filled in circles represent the five different groups of electrodes averaged together to form the 5 ROIs for ERP analysis. LAS, left anterior superior; RAS, right anterior superior; LPS, left posterior superior; RPS, right posterior superior; RFP, right fronto‐polar

Groups of electrodes were averaged together to form regions of interest (ROI; Figure 2), similar to what has been done by other researchers (Ally & Budson, 2007; Norman, Tepe, Nyhus, & Curran, 2008; Ross et al., 2015). Our analyses were focused on the left anterior superior (LAS), right anterior superior (RAS), left posterior superior (LPS), right posterior superior (RPS), and right fronto‐polar (RFP) ROIs. These ROIs were selected due to their relevance to old/new effects (Ally & Budson, 2007; Budson et al., 2005; Curran, 2000; Curran, Tepe, & Piatt, 2006; Norman et al., 2008; Rugg et al., 1998) and the late posterior negativity (LPN; Johansson & Mecklinger, 2003; Leynes & Kakadia, 2013; Leynes & Phillips, 2008; Rosburg, Mecklinger, & Johansson, 2011). The FN400 (300–500 ms poststimulus) is expected to appear at the LAS and RAS ROIs, whereas the late frontal old/new effect (1,000–1,500 ms poststimulus) should be observed in the RFP ROI. The LPS ROI is where the left parietal old/new effect (500–800 ms poststimulus) should be observed, whereas the late posterior negativity should be found in both the LPS and RPS ROI 1,000–1,500 ms poststimulus.

### 2.6 Spectral analysis preprocessing

Spectral analyses were run to examine oscillatory power during hits and correct rejections. For the spectral analyses, datasets for item memory were repreprocessed in EEGLAB. Repreprocessing was done due to the differences in standard preprocessing steps for ERP and oscillatory analyses, particularly the need to use the cleanest data possible for oscillatory analysis. Preprocessing included filtering the data from 1 to 100 Hz, rereferencing to the average signal, and artifact rejection. Data were epoched into the same −800 to 1,500 ms epochs as for the ERP analysis and sorted into hits and correct rejections bins. For the spectral analyses, artifact rejection was accomplished through EEGLAB's automatic epoch rejection function. EEGLAB's automatic epoch rejection function was set to detect and remove epochs that possessed voltage fluctuations of over 1,000 μV, as well as data deemed to be mathematically improbable, with this probability threshold set at five standard deviations. Upon completion of automatic epoch rejection, Infomax‐based independent component analysis (ICA; Bell & Sejnowski, 1995) was run. At this stage of data processing, datasets from two male participants belonging to the 10/10 homozygous DAT group experienced unresolvable errors related to the ICA decomposition. These errors prevented these two participants from being entered into EEGLAB's STUDY function for clustering and analyses. Therefore, these participants were dropped, leading to a total of 56 DAT participants (25 10/10 homozygous, 31 9R‐carrier) for oscillatory analyses. The resulting component activities were manually inspected, and epochs containing notable synchronous artifactual activity that failed to be separated by the initial ICA decomposition were manually marked and rejected. ICA was run again on the pruned data and ADJUST 1.1 (Mognon, Jovicich, Bruzzone, & Buiatti, 2011) was utilized to automatically remove noise components at the end of the second ICA. All independent components not deemed to be artifactual by ADJUST were source localized using the DIPFIT2 method (Oostenveld, Fries, Maris, & Schoffelen, 2011) based on a spherical 4 shell model.

### 2.7 Genotyping

Genomic DNA was isolated from saliva samples collected using a commercial product (Oragene^™^, DNAgenotek, Ottawa, ON, Canada). A common genetic variant of the DAT gene (*SLC6A3*) is a 40‐bp variable number tandem repeat (VNTR) sequence that repeats 9 or 10 times (Vandenbergh et al., 1992), with individuals possessing a copy of the 9‐repeat VNTR (9‐carriers) displaying decreased DAT expression (Fuke et al., 2001; Heinz et al., 2000) and increased synaptic dopamine (Heinz et al., 2000) compared to 10/10 homozygotes. This 40 bp *DAT1* VNTR (rs28363170) was genotyped as described in Haberstick et al. (2014). During genotyping, roughly one‐third of the samples (18 random, six for one or more genotype assignments) were regenotyped (a new PCR and fragment analysis) resulting in two previously failed samples to be assigned genotypes. All other samples were consistent between runs. *DAT* groups were split according to whether the variable nucleotide tandem repeat (VNTR) sequence that influences DAT expression repeated 9 or 10 times (Fuke et al., 2001). Participants that were heterozygous or homozygous for the 9‐repeat version of the allele (i.e., 9/9 or 9/10) were placed in one group, whereas participants homozygous for the 10‐repeat VNTR were placed in the other. The DAT genotype frequencies were distributed according to the Hardy–Weinberg Equilibrium (9.1% 9/9, 42.1% 9/10, 48.8% 10/10).

### 2.8 Behavioral analysis

Reaction time and accuracy were compared separately with 2 × 2 repeated measures ANOVAs. Item hit and correct rejection accuracy and reaction time were compared across *DAT* (10/10 homozygous and 9‐carrier) groups. Where appropriate, post hoc tests comprised of paired samples and independent samples *t* tests were run. Confidence ratings were used to extract ROC curves in order to determine response sensitivity and response bias without assuming old and new strength distributions have equal variance. Response sensitivity measured using *d*_a_, and response bias measured with *c*_a_ were compared between *DAT* groups (10/10 homozygotes and 9‐carriers) with independent samples *t* tests. For all behavioral analyses, the *p*‐value was set to *p* = .05 for statistical significance.

### 2.9 ERP analysis

ERP data during item memory retrieval were analyzed in five ROIs (LAS, RAS, LPS, RPS, and RFP) at three time frames poststimulus presentation: 300–500, 500–800, and 1,000–1,500 ms. Hit and CR mean ERP amplitudes were averaged in each ROI in all three time points of interest. Using SPSS 22.0 (SPSS Inc., Chicago, IL; RRID: SCR_002865), repeated measures ANOVAs were conducted to investigate any differences between hit and CR mean amplitudes within the four ROIs as a function of *DAT* groups. In the 300–500 ms poststimulus time frame, two separate 2 × 2 × 2 repeated measures ANOVAs were conducted with hemisphere (LAS and RAS), condition (hit and CR), and *DAT* (10/10 homozygous and 9R allele carrier) group as factors. Separate 2 × 2 repeated measures ANOVAs with condition (hit and CR) and *DAT* (10/10 homozygous and 9R allele possessing) group as factors were run for the LPS ROI 500–800 ms poststimulus and in the RFP 1,000–1,500 ms poststimulus. To analyze the LPN, 2 × 2 × 2 repeated measures ANOVAs were conducted for the 1,000–1,500 ms time frame, with hemisphere (LPS and RPS), condition (hit and CR), and *DAT* (10/10 homozygous and 9R allele carrier) group as factors. When appropriate, post hoc tests comprised of paired samples *t* tests comparing mean amplitudes during hits and CR within each group and independent samples *t* tests directly comparing genetic group differences were run. The *p*‐value was set to *p* = .05 for statistical significance for all ERP analyses.

### 2.10 Oscillatory analyses

EEGLAB's STUDY function was used to compare the oscillatory activity between hit and correct rejections, as well as the influence of DAT group (9R‐carrier and 10/10 homozygous) on oscillatory correlates of recognition memory. Event‐related spectral perturbations (ERSPs) and scalp maps were calculated for each of the independent components involved in oscillatory analyses. Data were converted to the time frequency domain in roughly 9 ms steps across 30 log‐spaced frequencies from 4 to 50 Hz using a Morlet wavelet transformation (Delorme & Makeig, 2004) from 522 ms precue to 1,218 ms postcue for each trial. The beginning and ending boundaries of the −800 to 1,500 ms epochs were cut to account for boundary artifacts introduced by wavelet transformation. The length of the wavelets increased from 2 cycles at 4 Hz to 12.8 cycles at 50 Hz. Component clustering was then utilized in order to identify sets of related independent components within and across participants. Prior to component clustering, the number of components included for analysis were automatically preselected, with only components with dipole model residual variance of less than 15% included for component clustering. Independent component clustering within and across participants was performed using *k*‐means with dipoles as the defining criterion to generate 15 independent component clusters. Independent components that did not fall within three standard deviations from any cluster centroid were excluded as outlier components. Eight component clusters located in frontal and parietal regions with at least 30 contributing participants were observed due to these regions’ relevance in memory processes. Statistical analyses were performed to determine whether main effects of condition (hits vs. correct rejections) or DAT group (9R‐carrier vs. 10/10 homozygotes) on oscillatory activity existed, along with the presence of a potential interaction between the two. EEGLAB's permutation‐based statistics function was utilized and set to 1,000 permutations, and the *p*‐value for statistical significance was set to *p* = .05 using an FDR correction for multiple comparisons.

## 3 RESULTS

### 3.1 DAT behavioral results

**Figure 3:**
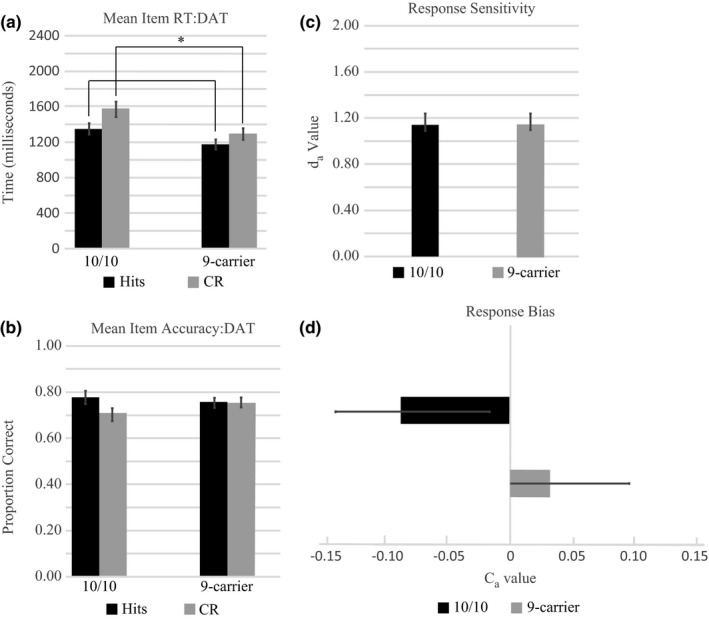
Item memory behavioral results. (a) Mean reaction time during hits (black bars) and correct rejections (gray bars) as a function of DAT polymorphism in the item recognition task. 10/10 homozygotes display significantly slower correct rejection times and a trend toward significantly slower hit reaction times compared to 9R‐carriers. (b) Item memory accuracy as a function of DAT polymorphism. Black bars illustrate the proportion of hits, whereas gray bars illustrate the proportion of correct rejections. The proportion of hits was not significantly different than the proportion of correct rejections, and no significant differences as a function of DAT group were observed. (c) Response sensitivity (*d*_a_) as a function of DAT polymorphism. The black bar illustrates response sensitivity as a function of the 10/10 DAT group, whereas the gray bar illustrates response sensitivity of the 9R‐carrier group. There were no observed differences in response sensitivity as a function of DAT polymorphism. (d) Response bias (*c*_a_) as a function of DAT polymorphism. The black bar illustrates response sensitivity as a function of the 10/10 DAT group, whereas the gray bar illustrates response sensitivity of the 9R‐carrier group. No significant differences in response bias as a function of DAT polymorphism were observed. Error bars represent standard error of the mean. *Represents significance at *p* ≤ .05

The 2 (condition) × 2 (*DAT* group) repeated measures ANOVA comparing reaction time in the item memory task revealed main effects of condition (*F*_1,56_ = 48.82, *p* < .0001, partial η^2^ = 0.47) and *DAT* group (*F*_1,56_ = 5.30, *p* = .03, partial η^2^ = 0.09). A significant interaction between condition and *DAT* group was observed (*F*_1,56_ = 4.23, *p* = .05, partial η^2^ = 0.07, Figure 3a). Independent samples *t* tests indicated that there were significant differences in CR reaction times (*t*_56_ = 2.49, *p* = .02, Cohen's *d *=* *0.65) between 10/10 homozygotes and 9R‐carriers, and a trend toward a significant difference in hit reaction time (*t*_56_ = 1.93, *p* = .06, Cohen's *d *=* *0.51) between the two DAT groups (Table 1). Paired samples *t* tests comparing reaction times during hits and CR trials within 10/10 homozygotes and 9R‐carriers displayed significant differences in both *DAT* groups. Mean hit reaction times for 10/10 homozygotes were significantly faster than CRs (*t*_26_ = 4.87; *p* < .0001, Cohen's *d *=* *0.94), whereas 9R‐carriers also displayed significantly faster mean hit reaction times compared to correct rejections (*t*_30_ = 5.28; *p* < .0001, Cohen's *d *=* *0.95). The size of the difference between hit and correct rejection reaction times are similar between 10/10 homozygotes than 9R‐carriers (10/10 homozygotes Cohen's *d *=* *0.94; 9R‐carriers Cohen's *d *=* *0.95). These reaction time results indicate that hit responses are faster than correct rejections in both genetic groups and that DAT genetic polymorphisms affect the speed at which these responses occur, with 10/10 homozygotes displaying slower hit and correct rejection response times compared to 9‐carriers.

**Table 1:** Mean reaction time for hits and correct rejections as a function of DAT polymorphism (in ms)

	10/10 homozygotes	9‐carrier
Hits	1,351.17	1,188.51
SEM (hits)	63.25	56.17
Correct rejections	1,572.35	1,309.14
SEM (CR)	84.85	65.48

The 2 (hits vs. correct rejections) × 2 (*DAT* group) repeated measures ANOVA comparing the proportion correct in the item memory task revealed no main effects of condition (*F*_1,56_ = 2.48, *p* = .12, partial η^2^ = 0.04) or *DAT* group (*F*_1,56_ = 0.31, *p* = .58, partial η^2^ = 0.01,). No significant interaction between condition and *DAT* group was observed (*F*_1,56_ = 1.72, *p* = .19, partial η^2^ = 0.03, Figure 3b). Independent samples *t* tests run to determine whether there was a difference between response sensitivity (*d*_a_) or response bias (*c*_a_) as a function of *DAT* group (*d*_a_: *t*_56_ = 0.04, *p* = .97, Cohen's *d *=* *0.01; *c*_a_: *t*_56_ = 1.48, *p* = .15, Cohen's *d *=* *0.39) revealed no significant differences (Figure 3c,d).

### 3.2 ERP results

#### 3.2.1 FN400 during item memory

**Figure 4:**
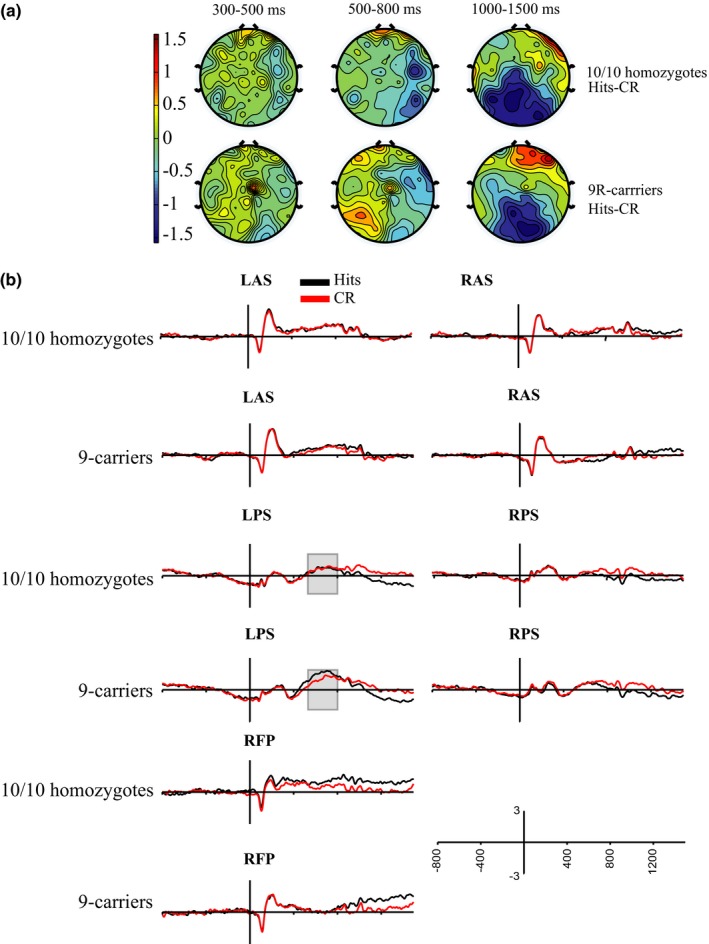
Item memory ERP results. (a) Topographical maps representing the distribution of ERP differences between hits and CRs (hits minus correct rejections) for 10/10 homozygotes (top row) and 9R‐carriers (bottom row) across the 300–500 ms (left), 500–800 ms (middle), and 1,000–1,500 ms (right) time frames. (b) Averaged group ERP waveforms in anterior and posterior ROIs. Averaged ERP waveforms from −800 to 1,500 ms poststimulus presentation (*y* axis cross at 0 ms) in the left anterior superior (LAS, top left panels), right anterior superior (RAS, top right panels), left posterior superior (LPS, middle left panels), right posterior superior (RPS, middle right panels) and right fronto‐polar (RFP, bottom left panels) ROIs for hits (black) and CRs (red) during item memory. 10/10 homozygote ERPs are represented in the first, third, and fifth rows, whereas 9‐carriers are represented in the second, fourth, and sixth rows. The gray boxes highlight the 500–800 ms timeframe in the LPS ROI in which 10/10 homozygotes and 9‐carriers show significant differences in the old/new effect

The 2 (hemisphere) × 2 (hits vs. correct rejections) × 2 (*DAT* group) repeated measures ANOVA examining mean ERP amplitude in the left anterior superior (LAS) and right anterior superior (RAS) ROIs at 300–500 ms poststimulus revealed a main effect of hemisphere (*F*_1,56_ = 9.36, *p* = .003, partial η^2^ = 0.14). However, main effects of condition (*F*_1,56_ = 0.36, *p* = .55, partial η^2^ = 0.01, Figure 4b) and *DAT* group (*F*_1,56_ = 1.97, *p* = .17, partial η^2^ = 0.03, Figure 4b) were not significant, nor was the condition × hemisphere × *DAT* group interaction (*F*_1,56_ = 0.08, *p* = .78, partial η^2^ = 0.001). All other possible interactions between hemisphere, condition, and *DAT* group (condition × *DAT* group; hemisphere × *DAT* group; condition × hemisphere) were also not significant.

#### 3.2.2 Parietal old/new effect during item memory

**Figure 5:**
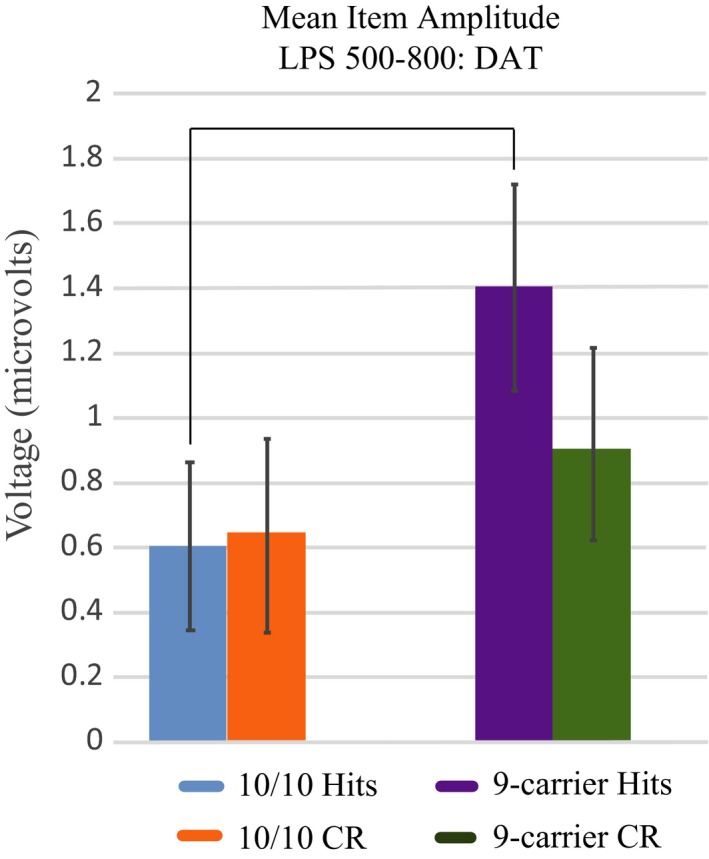
Bar graph illustrating ERP amplitude differences in LPS 500–800 ms post‐stimulus presentation during item memory. The standard error of the means are designated with error bars. Average ERP amplitudes for 10/10 homozygotes (10/10 hits, blue) and 9‐carriers (9‐carrier hits, purple) during item memory hits. Correct rejections are represented in orange for 10/10 homozygotes and in green for 9‐carriers. The ERP amplitude between 10/10 homozygote and 9‐carrier hits suggests a trend toward a significant difference, with 10/10 homozygotes displaying decreased ERP hit amplitude

The 2 (hits vs. correct rejections) × 2 (*DAT* group) repeated measures ANOVA conducted for the left posterior superior ROI at 500–800 ms poststimulus presentation revealed a trend toward a significant main effect of condition (*F*_1,56_ = 3.71, *p* = .06, partial η^2^ = 0.06), and a significant interaction between condition and *DAT* group (*F*_1,56_ = 4.86, *p* = .03, partial η^2^ = 0.08). A paired samples *t* test comparing mean ERP amplitudes between hit and CR trials within 9R‐carriers revealed the presence of the old/new effect (*t*_30_ = 3.89, *p* = .001, Cohen's *d *=* *0.70). Mean ERP amplitude in 9R‐carrier participants for hit trials was significantly larger than CR trials. Participants that were 10/10 homozygotes did not display the old/new effect as there was no significant difference between mean ERP amplitude during hit trials compared to CR trials (*t*_26_ = 0.16, *p* = .88, Cohen's *d *=* *0.03, Figure 4b). Independent sample *t* tests indicated that there was no difference in CR mean amplitude between 10/10 homozygotes and 9R‐carriers (*t*_56_ = 0.65, *p* = .52, Cohen's *d* = 0.17, Figure 5). A trend toward a significant difference was observed when mean amplitude during hits was compared between *DAT* groups (*t*_56_ = 1.89, *p* = .07, Cohen's *d* = 0.49, Figure 5) suggesting that 10/10 homozygous participants do not show the left parietal old/new effect due to decreased mean ERP amplitudes during hit trials compared to participants carrying a 9R‐allele.

#### 3.2.3 Late frontal old/new effect during item memory

The 2 (hits vs. correct rejections) × 2 (*DAT* group) repeated measures ANOVA conducted for the right fronto‐polar ROI 1,000–1,500 ms poststimulus did not reveal a main effect of condition (*F*_1,56_ = 3.60, *p* = .06, partial η^2^ = 0.06) or any interaction between *DAT* group and condition (*F*_1,56_ = 0.10, *p* = .75, partial η^2^ = 0.002). Mean ERP amplitude for hit trials were not significantly higher than CR trials.

#### 3.2.4 Late posterior negativity during item memory

The late posterior negativity during the item memory task was examined with a 2 (hemisphere) × 2 (condition) × 2 (*DAT* group) repeated measures ANOVA conducted for the left posterior superior and right posterior superior ROIs 1,000–1,500 ms poststimulus presentation. The ANOVA revealed a main effect of condition (*F*_1,56_ = 26.02, *p* < .0001, partial η^2^ = 0.32). There were no observed main effects of hemisphere (*F*_1,56_ = 0.88, *p* = .35, partial η^2^ = 0.02) or *DAT* group (*F*_1,56_ = 0.44, *p* = .51, partial η^2^ = 0.01), and all possible interactions between hemisphere, condition, and *DAT* group (hemisphere × *DAT* group; condition × *DAT* group; hemisphere × condition; hemisphere × condition × *DAT* group) failed to reach significance.

### 3.3 Effects of DAT polymorphism on oscillatory power during item memory

**Figure 6:**
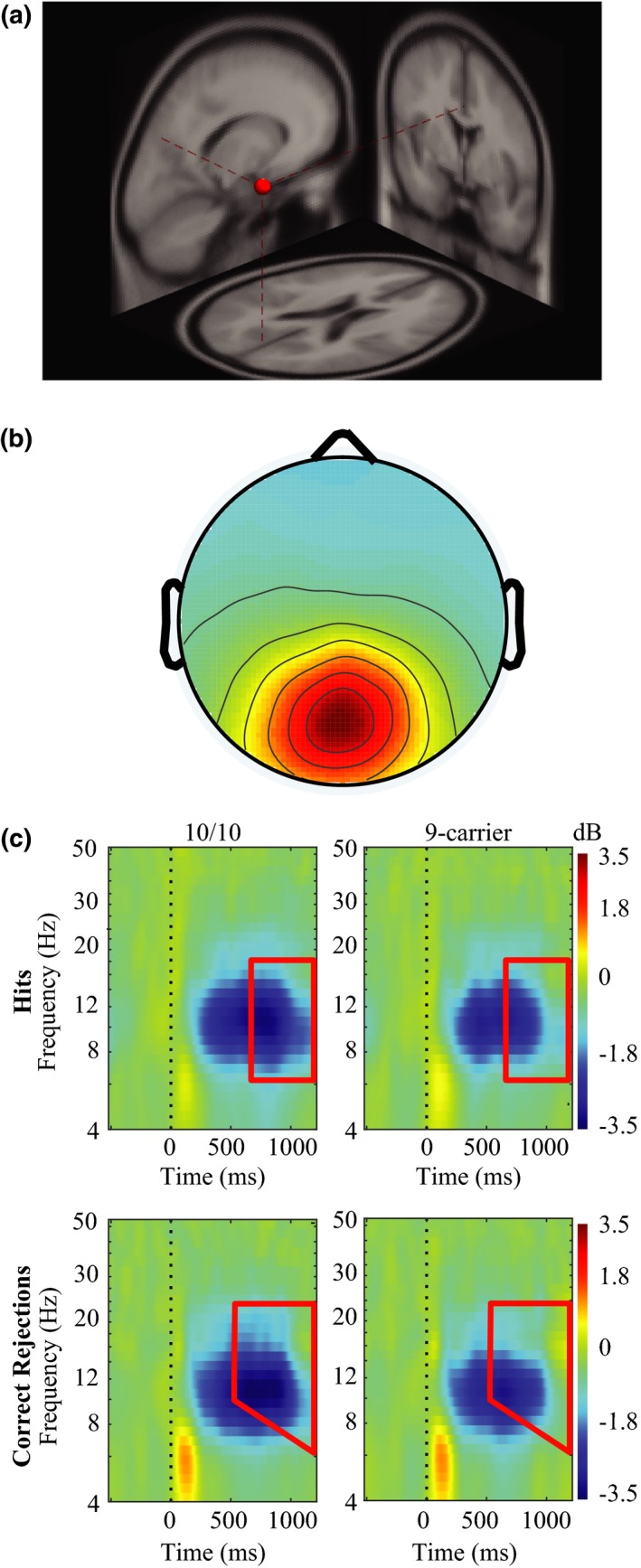
Event‐related spectral perturbation (ERSP) results for the midparietal component cluster. (a) Average dipole location for the midparietal component cluster. Dashed red lines indicate the average dipole location for the mid parietal component cluster when mapped onto a standardized brain model. (b) Corresponding scalp map for the midparietal component cluster. (c) ERSPs for the midparietal component cluster. The top row of graphs represents oscillatory power differences during hits and the bottom panel represents power differences during correct rejections in the midparietal component cluster, with 10/10 homozygous participants on the left and 9‐carriers on the right. Bounded red boxes represent areas of statistical differences between groups with 10/10 homozygotes showing decreased theta, alpha, and beta power during both hits and correct rejections

Postcomponent clustering, three distinct component clusters located in the midparietal region (40 participants, 80 independent components), midfrontal region (40 participants, 90 independent components), and left parietal region (34 participants, 57 independent components) displayed significant differences in oscillatory activity as a function of the *DAT* gene. The midparietal component cluster (Figure 6) showed a significant effect of DAT group on oscillatory power for both hits and correct rejections. Significant differences in hit oscillatory power were observed between 10/10 homozygotes and 9‐carriers in a frequency range from theta to early beta (5–18 Hz). Differences in theta band activity (4–8 Hz) were observed occurring from 740 to 1,108 ms poststimulus, whereas differences in alpha (8–12 Hz) and early beta band (13–18 Hz) activity were observed from roughly 740–1,218 ms postcue presentation. Analyses of correct rejection oscillatory activity revealed similar results, with significant differences in correct rejection oscillatory power observed between 10/10 homozygotes and 9‐carriers in a frequency range from theta to beta (6.5–26 Hz). Differences in theta band activity (6.5–8 Hz) were observed starting 845–1,218 ms postcue presentation, whereas differences in alpha (8–12 Hz) were observed occurring from 714 to 1,218 ms postcue presentation. Significant differences in beta oscillatory activity (13–26 Hz) occurred earlier during correct rejections, starting at 610 ms and ending 1,218 ms postcue presentation. These oscillatory differences during item memory hits and correct rejections appear to be driven by a larger and longer lasting decrease in oscillatory power in 10/10 homozygotes.

**Figure 7:**
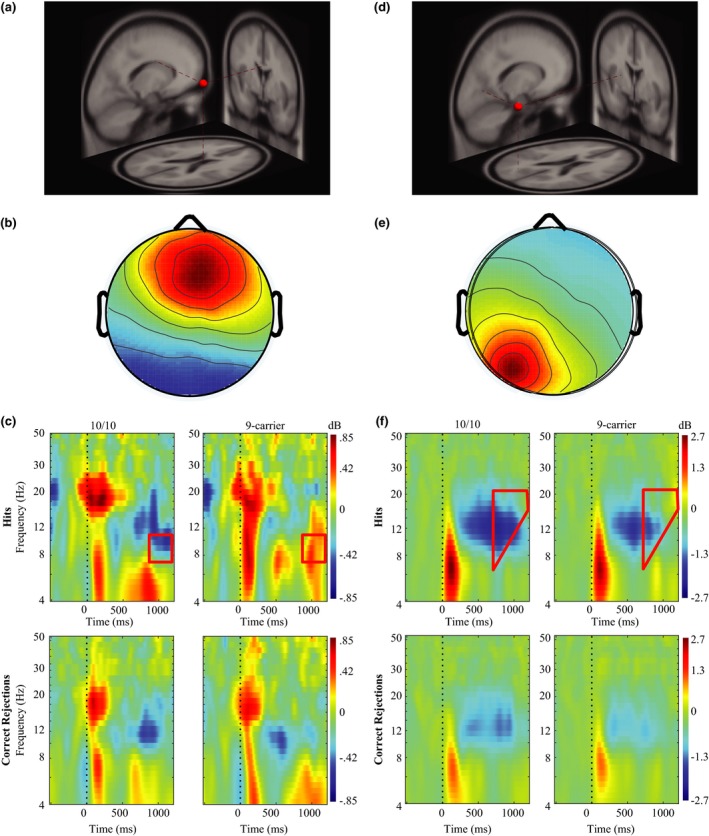
Event‐related spectral perturbation (ERSP) results for the midfrontal and left parietal component clusters. (a) Average dipole location for the midfrontal component cluster. Dashed red lines indicate the average dipole location for the midfrontal component cluster when mapped onto a standardized brain model. (b) Corresponding scalp map for the midfrontal component cluster. (c) ERSPs for the midfrontal component cluster. The top row of graphs represents oscillatory power differences during hits and the bottom panel represents power differences during correct rejections in the midparietal component cluster, with 10/10 homozygous participants on the left and 9‐carriers on the right. Bounded red boxes represent areas of statistical differences between groups with 10/10 homozygotes showing decreased alpha power during hits, but not correct rejections. (d) Average dipole location for the left parietal component cluster. Dashed red lines indicate the average dipole location for the left parietal component cluster when mapped onto a standardized brain model. (e) Corresponding scalp map for the left parietal component cluster. (f) ERSPs for the left parietal component cluster. The top row of graphs represents oscillatory power differences during hits and the bottom panel represents power differences during correct rejections in the left parietal component cluster, with 10/10 homozygous participants on the left and 9‐carriers on the right. Bounded red boxes represent areas of statistical differences between groups with 10/10 homozygotes showing decreased theta, alpha, and beta power during hits, but not correct rejections

The midfrontal component cluster displayed significant differences in oscillatory power between the *DAT* groups during item memory hits (Figure 7c). During item memory hits, 10/10 homozygotes display a significant decrease in alpha (8–11 Hz) occurring from 924 ms and lasting until 1,176 ms postcue presentation. Analyses of oscillatory activity during correct rejections in the midfrontal component cluster yielded no significant differences.

Much like the results observed in the midfrontal component cluster, significant differences in oscillatory power between DAT groups were observed for hits but not correct rejections in the left parietal component cluster. During item memory hits (Figure 7f), the left parietal component cluster displayed significant differences in theta to beta (5–24 Hz) oscillatory power. A brief period of significantly different theta activity (5–8 Hz) was observed occurring from 741 to 967 ms. Differences in alpha and beta oscillatory activity (8–24 Hz) between 10/10 homozygotes and 9‐carriers during item memory hits were observed to last for a longer period, with alpha band activity (8–12 Hz) significantly different from a period 705–1,177 ms postcue presentation and beta band activity (13–24 Hz) displaying significant differences from 697 to 1,218 ms postcue presentation. Much like the results displayed by the midparietal and midfrontal component clusters, these differences appear to be driven by a significant decrease in oscillatory power occurring in 10/10 homozygotes.

## 4 DISCUSSION

This study investigated how genetic polymorphisms in the dopamine transporter gene (*SLC6A3*) influences behavioral and electrophysiological correlates of recognition memory. Behaviorally, participants that are 10/10 homozygous display significantly slower hit and correct rejection response times compared to participants possessing a copy of the 9R allele. The results of this study indicate that participants homozygous for the 10R VNTR of the dopamine transporter gene display decreased hit amplitudes in the left posterior superior region of interest 500–800 ms poststimulus during item memory. Oscillatory analyses further reveal that 10/10 homozygotes display decreases in theta, alpha, and beta oscillatory power in a midparietal component cluster during hits and correct rejections. In contrast, analyses of a midfrontal and left parietal component cluster revealed 10/10 homozygotes display significant decreases in predominantly alpha and beta during hits, but not correct rejections. These results suggest that dopamine transporter genetic variation affects both ERP and oscillatory dynamics of memory retrieval, which may account for the significant increase in reaction times for both the correct recognition of old and new items during recognition memory. Together, the ERP and oscillatory results suggest that *DAT* may be involved in how individuals perform recognition memory.

### 4.1 DAT polymorphism and behavioral correlates of item memory retrieval

Our study results show significant differences in response times as a function of DAT polymorphism, with participants homozygous for the 10R VNTR displaying significantly slower reaction times for both hits and correct rejections compared to participants possessing a 9R allele. Increasing dopamine levels during item memory results in faster response times for both hits and correct rejections (Apitz & Bunzeck, 2013; Bunzeck et al., 2009; Eckart & Bunzeck, 2013), demonstrating a link between dopamine and recognition response times. Dopaminergic neurotransmission is partially regulated by the dopamine transporter, which serves to retrieve synaptic dopamine and return it to presynaptic neurons, terminating dopaminergic signaling. Participants homozygous for the 10R VNTR have increased DAT expression (Fuke et al., 2001), increased dopaminergic reuptake, and decreased synaptic dopamine (Heinz et al., 2000). With studies showing that altering dopamine levels changes recognition response time (Apitz & Bunzeck, 2013; Bunzeck et al., 2009; Eckart & Bunzeck, 2013), the decreased synaptic dopamine hypothesized to be occurring in 10/10 homozygotes may explain the increased hit and correct rejection reaction times displayed by 10/10 homozygous participants performing our item memory task.

### 4.2 DAT polymorphism and ERP correlates of recognition memory

Dopamine may contribute to recognition memory by modulating the level of information retrieved for postretrieval processing, with the dopamine transporter polymorphism playing a significant role in this modulation. ERP studies of recognition memory have identified several distinct neural correlates known as old/new effects, which are more positive ERP deflections for hits compared to correct rejections (Curran, 2000; Curran et al., 2006; Donaldson & Rugg, 1998; Friedman & Johnson, 2000; Rugg & Curran, 2007; Rugg et al., 1998; Vilberg & Rugg, 2007; Wilding & Rugg, 1996; Woodruff, Hayama, & Rugg, 2006). The left parietal old/new effect is associated with the amount of information retrieved (Vilberg & Rugg, 2007; Vilberg et al., 2006; Wilding, 2000). Specifically, Wilding (2000) show that the left parietal old/new effect is larger when retrieving more contextual details associated with an item. Additionally, Vilberg et al. (2006) found that the magnitude of the parietal old/new effect is larger when participants fully recollect available visual information compared to partial recollection using a Remember/Know task. Our results show that participants carrying a 9R‐allele display a robust left parietal/old new effect, along with greater mean hit amplitude than their 10/10 counterparts (Figure 4). Together, the finding that the magnitude of the left parietal old/new effect is related to amount of information retrieved and that 9R‐carriers show the left parietal old/new effect suggests that 9R carriers are accessing greater amounts of information to support their recognition judgments. In contrast, 10/10 homozygous participants fail to display the left parietal old/new effect. To ensure that this failure to display the left parietal old/new effect was not simply delayed to a time period after 800 ms, post hoc tests on mean ERP amplitudes in the left posterior superior ROI were conducted for the time period 800–1,000 ms postcue presentation. The presence of a potentially delayed old/new effect was not observed, and no differences between hit and correct rejection mean amplitudes were observed within DAT groups (10/10 homozygotes: *t*_26_ = 1.47, *p* = .15, Cohen's *d *=* *0.28; 9R‐carriers: *t*_30_ = 0.59, *p* = .56, Cohen's *d *=* *0.11). Thus, 10/10 homozygotes may have access to less information during the item memory task resulting in slowed performance. Combined with the finding that 10/10 homozygous participants have increased dopamine transporter expression (Fuke et al., 2001), which is associated with increased synaptic dopamine clearance (Heinz et al., 2000), our results suggest dopamine function may relate to controlling the amount of information available during recognition memory.

Though the 10/10 homozygous participants do not show a left parietal old/new effect and are slowed during task performance, they still are accurate at identifying items as old or new. Accuracy in the recognition memory task may be related to other ERP components associated with memory. Our study results show that the item memory task elicited no significant differences in the early frontal or late frontal old/new effects across participants, regardless of DAT polymorphic group. Due to the presence of a main effect of condition during the late posterior negativity, subsequent analyses were further conducted for this later ERP signature. Subsequent analyses on the late posterior negativity, a hypothesized ERP signature of evaluative cognitive control processes associated with retrieved contextual details (Johansson & Mecklinger, 2003), showed that the LPN was present in both DAT groups, suggesting that the LPN occurs during the item memory task regardless of DAT polymorphism. This pattern of ERP results may provide some rationale as to why accuracy is unaffected, whereas mean reaction times were affected by DAT polymorphism. Alongside absent early frontal and left parietal old/new effects, these results suggest the LPN component may be associated with item memory performance in 10/10 homozygotes.

### 4.3 DAT polymorphism and oscillatory correlates of item memory retrieval

Analyses of oscillatory activity in midparietal, midfrontal, and left parietal component clusters suggest that DAT polymorphisms affect the oscillatory dynamics associated with recognition memory. Differences between DAT polymorphisms in the midparietal component cluster occurred at roughly the same time periods and frequency ranges for both hits and correct rejections (Figure 6), with 10/10 homozygous participants displaying decreases in theta, alpha, and beta power in both conditions. Differences in retrieval related oscillatory activity were also evident in the midfrontal and left parietal component clusters during hits (Figure 7), as 10/10 homozygotes displayed a significant decrease in alpha oscillatory power in the midfrontal component cluster and significant decreases in alpha and beta power in the left parietal component cluster compared to participants possessing a 9R‐allele. These combined results suggest that DAT polymorphism affects the oscillatory dynamics of correctly identifying old and new items, with these various oscillatory dynamics potentially reflective of different retrieval strategies.

Increased alpha and beta desynchronization in 10/10 homozygotes may allow for accurate recognition of new or old items despite the lack of a parietal old/new effect. Alterations in alpha and beta power have been linked to memory processes (Fell et al., 2008; Fellner et al., 2013; Hanslmayr et al., 2009, 2011; Sederberg et al., 2007; Waldhauser et al., 2012; Weiss & Rappelsberger, 2000). Specifically, the desynchronization hypothesis postulates that decreases in alpha and beta power, resulting in desynchronization of neural ensembles, are related to memory retrieval (Düzel et al., 2003; Hanslmayr et al., 2012; Khader & Rösler, 2011; Spitzer, Hanslmayr, Opitz, Mecklinger, & Bäuml, 2008), with larger decreases in alpha and beta power associated with the retrieval of more information (Khader & Rösler, 2011). Neurons that fire synchronously convey less information compared to neurons that fire asynchronously (Hanslmayr et al., 2012; Schneidman et al., 2011). Therefore, alpha and beta desynchrony may allow for a small network of neurons to generate an infinite number of neural firing patterns allowing a vast amount of information to be sent from a local neural assembly (Hanslmayr et al., 2012). Participants homozygous for the 10‐repeat allele display significantly decreased alpha/beta power during hits in midparietal, midfrontal, and left parietal component clusters (Figures 6 and 7), with these decreases lasting for a longer period of time compared to 9R‐carriers. These decreases of alpha/beta power coincide with prior results showing decreases in alpha/beta power during memory retrieval (Düzel et al., 2003; Khader & Rösler, 2011; Spitzer et al., 2008). Our experiment extends these findings by showing that polymorphisms of the dopamine transporter gene affect the amount of desynchronization occurring during a memory retrieval task, with 10/10 homozygotes showing greater, longer lasting desynchronization compared to 9R‐carriers. 10/10 homozygotes fail to display the parietal old/new effect, an ERP marker of contextual information retrieval, along with displaying significantly slower reaction times for hits. The extended period of alpha/beta desynchrony 10/10 homozygotes show may reflect a method necessary to successfully perform the memory task. While 9R‐carriers are able to retrieve the necessary information within a shorter amount of time, 10/10 homozygotes continue to utilize neural communication via alpha/beta desynchronization to obtain the necessary information needed for making correct judgments of previously encountered items. This extended period of neural communication 10/10 homozygotes utilize may allow for task accuracy to be maintained, at the expense of slowed reaction times.

Genetic variation in *DAT* also affected how the identification of new items might occur. Correct rejections involve the ability to correctly identify a previously unencountered item as new, and the midparietal component cluster displays a significant, longer lasting decrease in alpha/beta oscillatory power during correct rejections for 10/10 homozygotes (Figure 6c). While increased alpha and beta desynchrony during item hits for 10/10 homozygotes may be reflective of increased information transmission regarding the item presented, the increased desynchrony occurring during correct rejections may be reflective of greater amounts of information transmitted regarding the results of a memory search process undertaken for a new item. Thus, the decreased power displayed by 10/10 homozygotes in the midparietal component cluster may suggest that 10/10 homozygotes send more information when making judgments of correct rejections compared to 9R‐carriers, much like 10/10 homozygotes’ actions during hits. Much like the midparietal component cluster's activity during hits, the extended period of neural communication utilized by 10/10 homozygotes may result in the occurrence of accurate correct rejection judgments at the expense of slowed reaction times.

Increased theta desynchrony observed in 10/10 homozygotes in the midparietal component cluster during both hits and correct rejections may be related to attentional processes underlying successful recognition. Previous studies utilizing functional MRI have identified increased activity in the parietal cortex during episodic memory retrieval (for reviews, see: Cabeza, Ciaramelli, Olson, & Moscovitch, 2008; Cabeza et al., 2011; Ciaramelli, Grady, & Moscovitch, 2008; Donaldson, Wheeler, & Peterson, 2009; Hutchinson, Uncapher, & Wagner, 2009; Olson & Berryhill, 2009; Wagner, Shannon, Kahn, & Buckner, 2005), with the parietal cortex displaying increased BOLD activity for old items compared to new items. The attention to memory model (AtoM) proposed by Cabeza et al. (2008) suggests that activity in the dorsal regions of the parietal cortex mediate top‐down attentional processes guided by an individual's goals, whereas activity in the ventral regions of the parietal cortex serve to signal bottom‐up attentional processes reflective of the need to change attentional focus after relevant memories have been successfully retrieved. While decreases in theta oscillatory activity may be related to the degree cognitive control processes are engaged (Cavanagh & Frank, 2014; Cooper, Darriba, Karayanidis, & Barcelo, 2016; van Driel, Sligte, Linders, Elport, & Cohen, 2015; González‐Villar & Carrillo‐De‐La‐Peña, 2017; Sauseng et al., 2006), decreased theta oscillatory activity over posterior parietal brain regions has also been related to attention, with a recent study performed by Friese et al. (2016) showing increased theta desynchronization when participants were required to attend to stimuli. Our study reveals significant differences in theta power between 9‐carriers and 10/10 homozygotes in a midparietal component cluster during a memory retrieval task, a result that suggests that DAT genetic polymorphisms affect attentional processes underlying successful memory retrieval. The presence of decreased theta power in the midparietal component cluster for 10/10 homozygotes for both hits and correct rejections suggests that 10/10 homozygotes may be utilizing increased top‐down or bottom‐up attentional processes to properly identify items as new or old.

### 4.4 Limitations

Our current study describes how individual differences in recognition memory are affected by genetic variation in the dopamine transporter gene by analyzing the behavioral, ERP, and oscillatory correlates of item memory retrieval. As we were focused on memory retrieval, no EEG data during the encoding process were recorded. Therefore, we cannot rule out that differences during encoding between our DAT groups could explain the differential EEG and behavioral results we observed. Future studies examining encoding differences should explore this issue. Additionally, Chabris et al. (2012) conclude that studies attempting to establish relationships between SNPs (single nucleotide polymorphisms) and cognitive abilities may be underpowered, requiring large participant numbers. Our study utilizes a relatively small sample of 58 participants, and this small sample size may account for our inability to find significant differences in item memory accuracy between our 9R‐carrier and 10/10 homozygote groups. However, our study displays moderate to large effect sizes regarding differences in both hits (Cohen's *d *=* *0.51) and correct rejections (Cohen's *d *=* *0.65) between 10/10 homozygotes and 9R‐carriers reaction times. The main ERP effect observed within our study was the interaction between condition and DAT group for the parietal old/new effect and the parietal old/new effect in the 9R‐carriers displays a large Cohen's *d* of 0.70, whereas the parietal old/new effect in 10/10 carriers reveals a Cohen's *d* of 0.03. This difference in effect size between 9R‐carriers and 10/10 homozygotes suggests that the lack of a parietal old/new effect in 10/10 homozygotes is a robust finding.

## 5 CONCLUSION

Our study aims to further understand individual differences in recognition memory by describing the effect dopamine transporter genetic variation has on both behavioral and electrophysiological correlates of recognition memory. Our results show that dopamine transporter genetic variation affects mean ERP amplitudes over left parietal scalp locations, with 10/10 homozygotes, who show increased DAT expression (Fuke et al., 2001), showing no left parietal old/new effect alongside significantly increased reaction times for hits and correct rejections. Oscillatory results show that a midparietal component cluster shows decreased theta, alpha, and beta power for hits and correct rejections in 10/10 homozygotes. Midfrontal and left parietal component clusters displayed decreased alpha/beta power in 10/10 homozygotes during recognition of old items, but not during the identification of items as new. The left parietal old/new effect is associated with the amount of information retrieved (Vilberg & Rugg, 2007; Vilberg et al., 2006; Wilding, 2000) and decreases in alpha and beta power have been associated with the increased transmission of information (Hanslmayr et al., 2012; Khader & Rösler, 2011). Therefore, our study suggests that individuals who have increased dopamine transporter expression may rely on the increased transmission of information in order to obtain the necessary information to make accurate object identifications, a process that results in slowed recognition.

## CONFLICT OF INTEREST

The authors declare no conflict of interest.

## AUTHOR CONTRIBUTIONS

All authors contributed extensively to the work presented in the paper. Erika Nyhus designed the research study, collected the data, and performed the initial behavioral and EEG analyses. Tim Curran helped Erika Nyhus with the study design. Andrew Smolen genotyped all of the study participants. Paolo Medrano performed the remaining behavioral analyses, processed the ERP and oscillatory data, and wrote the manuscript. Robert Ross aided Paolo Medrano with analyzing the behavioral and EEG data, along with editing the manuscript.
